# AP-1/σ1A and AP-1/σ1B adaptor-proteins differentially regulate neuronal early endosome maturation via the Rab5/Vps34-pathway

**DOI:** 10.1038/srep29950

**Published:** 2016-07-14

**Authors:** Ermes Candiello, Manuel Kratzke, Dirk Wenzel, Dan Cassel, Peter Schu

**Affiliations:** 1 Georg-August University Göttingen, Department for Cellular Biochemistry, Humboldtallee 23, D-37073 Göttingen, Germany; 2 Electron microscopy, Max-Planck-Institut for Biophysical Chemistry, Am Fassberg 11, D-37077 Göttingen, Germany; 3 Israel Institute of Technology, Department Biology, Haifa 32000, Israel

## Abstract

The σ1 subunit of the AP-1 clathrin-coated-vesicle adaptor-protein complex is expressed as three isoforms. Tissues express σ1A and one of the σ1B and σ1C isoforms. Brain is the tissue with the highest σ1A and σ1B expression. σ1B-deficiency leads to severe mental retardation, accumulation of early endosomes in synapses and fewer synaptic vesicles, whose recycling is slowed down. AP-1/σ1A and AP-1/σ1B regulate maturation of these early endosomes into multivesicular body late endosomes, thereby controlling synaptic vesicle protein transport into a degradative pathway. σ1A binds ArfGAP1, and with higher affinity brain-specific ArfGAP1, which bind Rabex-5. AP-1/σ1A-ArfGAP1-Rabex-5 complex formation leads to more endosomal Rabex-5 and enhanced, Rab5^GTP^-stimulated Vps34 PI3-kinase activity, which is essential for multivesicular body endosome formation. Formation of AP-1/σ1A-ArfGAP1-Rabex-5 complexes is prevented by σ1B binding of Rabex-5 and the amount of endosomal Rabex-5 is reduced. AP-1 complexes differentially regulate endosome maturation and coordinate protein recycling and degradation, revealing a novel molecular mechanism by which they regulate protein transport besides their established function in clathrin-coated-vesicle formation.

The ubiquitous AP-1 complex consists of four adaptin subunits. γ1 and β1 bind clathrin and ‘accessory’ proteins for vesicle formation, whereas μ1A and σ1A bind the cargo proteins for AP-1 clathrin-coated-vesicles (CCV)^1^,^2^. The μ1A subunit is also involved in the regulation of AP-1 membrane-cytoplasma recycling^3^,^4^. The σ1B isoform is 87% identical to σ1A. Although both bind the same cargo proteins with di-leucine-based sorting motifs, certain cargo proteins like sortilin^5^,^6^,^7^,^8^,^9^, are exclusively bound by σ1B. AP-1/σ1A is essential for brain development, whereas AP-1/σ1B takes part in synaptic vesicle recycling^7^,^10^. Humans with mutations in σ1A die post-natally, whereas the zebrafish ‘knock-down’ is viable^11^. σ1A −/− mice die *in-utero* and the few who are born die perinatally (Schu, unpublished). If AP-1/σ1A and AP-1/σ1B are absent, as in μ1A ‘knock-out’ mice, embryos have hemorrhages in the ventricles and the spinal canal and die at day 13.5 *in-utero*^10^. Thus, AP-1 complexes are indispensable for neuronal development and function, but the essential functions they fulfil are not well understood.

Neurotransmission is mediated by the fusion of synaptic vesicles (SV) with the presynaptic plasma membrane. SV have to be efficiently recycled to prevent depletion of the SV pool and the fusion site has to be cleared from SV proteins^12^ to ensure proper neurotransmission. Membrane and SV protein retrieval is mediated by clathrin-independent and clathrin-dependent pathways. Clathrin-independent pathways are the fastest pathways, operating within milliseconds and up to seconds, while clathrin-dependent pathways are slower and require several seconds to be completed^13^. In clathrin-dependent SV protein endocytosis CCV formation at the plasma membrane requires the AP-1-homologous AP-2 complex and additional vesicle coat proteins. After their uncoating, these endocytotic vesicles can fuse with early endosomes, from which SV are reformed in a AP-1-dependent manner^7^,^13^,^14^. A function of these early endosomes in the SV recycling route has not yet been demonstrated, but the impairment of endosomal SV protein sorting by σ1B-deficiency and the severe mental retardation demonstrate its importance for neurotransmission. σ1B is encoded on the X-chromosome in humans and mice. σ1B deficiency in humans leads to severe mental retardation and a delay of starting to walk. σ1B −/− mice develop the same phenotypes as the human patients, demonstrating conserved molecular functions. These animals have severely impaired spatial memory and learning, they are hypoactive and their motor coordination is impaired^7^,^15^.

Absence of AP-1/σ1B from pre-synapses leads to changes in diverse vesicular protein transport pathways. Reformation of SV during recycling is slower and incomplete, leading to a reduction in SV numbers, whereas phosphatidylinositol-3-phosphate (PI-3-P)-positive early endosomes accumulate. The synthesis of multivesicular body (MVB) late endosomes is up-regulated and protein levels of about one third of SV proteins are reduced, indicating enhanced degradation of SV proteins via the MVB pathway. Very surprisingly, these σ1B −/− synapses contain more of the endocytotic AP-2 CCV^7^,^16^.

These alterations in early endosome maturation and protein sorting in σ1B −/− synapses indicated to us that endosomal AP-1/σ1A and AP-1/σ1B complexes might take part in the regulation of MVB endosome formation. Analysing the σ1 dependence of these membrane dynamics we unraveled a novel molecular mechanism by which AP-1/σ1B and AP-1/σ1A regulate protein sorting, which does not involve the formation AP-1 CCV. We demonstrate in this study that they regulate synaptic early endosome membrane dynamics, enabling protein transport into an endolysosomal degradative pathway. A direct sorting function of AP-1 for SV proteins into SV and in SV reformation, as indicated by the ‘knock-out’ phenotype, remains to be demonstrated. Here we show that MVB pathway activation involves the σ1A-mediated formation of a novel Rab5-activating complex: AP-1/σ1A/ArfGAP1/Rabex-5. ArfGAP1 is the GTPase that activates Arf1^GTP^. Complex formation is most efficient with the brain-specific isoform of ArfGAP1. Rabex-5 is a Rab5 GDP-GTP exchange factor. Rab5 activates the PI 3-kinase Vps34 (PI3KC3), whose activity is essential for MVB formation^16^,^17^,^18^,^19^. σ1B binds Rabex-5, preventing ArfGAP1 binding and thus formation of the stable tripartite complex. Here we show that σ1A and σ1B AP-1 complexes, besides their longknown functions in protein sorting and CCV formation, coordinate SV protein recycling and SV protein degradation pathways. Our data support a model, in which AP-1 complexes regulate SV protein degradation via the MVB pathway by controlling early endosome maturation and by directing the degradation of selected SV proteins. This would enable the synthesis of SV with a modified protein composition and thus altered properties in neurotransmission. Also in Alzheimer’s and Parkinson’s diseases is the sorting of endosomal proteins disturbed and thus these novel AP-1 functions could also be important for the progression of these diseases.

## Results

### Vps34 is hyperactivated on σ1B −/− endosomes

**Figure 1:**
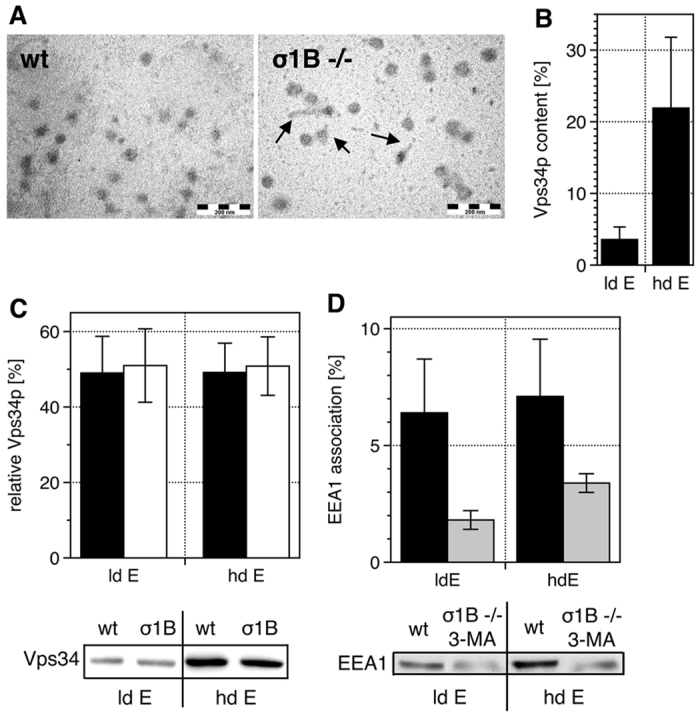
Synaptosomal distribution and activity of the PI3-kinase Vps34. Experiments were repeated with extracts from individual animals. (**A)** EM images of early endosome-containing density gradient fractions. Arrows indicate membrane dynamics of endosomes isolated from σ1B −/− brains. (**B)** Vps34 detected in low-density (ld) and high-density (hd) gradient fractions from wt brains enriched early (ld) and late (hd) endosomes, is expressed as % of Vps34 detected in all fractions (Σ = 100%) (n = 5; mean and s.d.). (**C)** σ1B deficiency does not alter the Vps34 content of these endosomes: wt (filled bars), σ1B −/− (open bars) (n = 5; mean and s.d.). (**D)** σ1B deficiency causes the redistribution of 23 ± 4 and 16.7 ± 4 of EEA1 into the early (id) and late (hd) endosome fractions of the gradient due to their increase in PI-3-P (see Kratzke *et al.*^16^ and Fig. 5). Addition of the specific Vps34 inhibitor 3-MA blocks PI-3-P formation, demonstrated by the reduced EEA1 membrane association: wt (filled bars), σ1B −/− plus 3-MA (grey bars) (n = 3; mean and s.d.). Representative Western blots are shown in (**C**,**D**).

Previous data show that synapses of σ1B −/− mice have enlarged endosomes and contain two times more PI-3-P than endosomes from wt mice. Also the late endosome MVB pathway, which depends on the PI 3-kinase Vps34, is upregulated in synapses of σ1B −/− mice^7^,^16^. These data indicate, that AP-1/σ1A and AP-1/σ1B may take part in the regulation of early endosome maturation into MVB endosomes via the regulation of Vps34-activity. In order to analyse such a molecular mechanism *in-vitro*, we first tested whether the enlarged endosomes seen in EM images *in-vivo* can still be found after the 5 h density centrifugation required for their isolation. Vesicles with tubules are readily detected in the early endosome (low-density, ld) density gradient fraction from σ1B −/−, but not in fractions from wt brains (Fig. 1A). Vps34 is bound to these early and late endosomes (high-density, hd) (Fig. 1B), but endosomes from σ1B −/− mice do not contain more Vps34 than wt endosomes (Fig.1C), indicating increased Vps34 specific activity. To test whether Vps34 activity is indeed up-regulated, we added the specific inhibitor 3-methyl adenine (3-MA)^20^. Endosomal PI-3-P levels of wt and σ1B −/− endosomes were compared by determining the amount of EEA1 bound to these endosomes via its PI-3-P-specific FYVE-domain (Fig.1D). EEA1 is not a presynaptic, or apical, protein and will bind to these endosomes only after cell lysis^16^,^21^,^22^,^23^,^24^. In the Kratzke *et al.*^16^ publication, we published the data showing that 23.2 ± 4.4 and 16.7 ± 3.1% of the loaded EEA1 is associated with the early endosome (ld) and late endosome (hd) enriched density gradient fractions. Endosomes from wt mice contain only 6.2 ± 2.5 and 7 ± 2.5% of the loaded EEA1. We performed experiments in parallel at the time adding 3-MA (5 mM) to the extracts from σ1B −/− neurons. Addition of the Vps34 PI 3-kinase inhibitor normalised EEA1 binding and reduced it even below wt levels (Fig. 1D; see also final result chapter and Kratzke *et al.*)^16^. Thus the increase in PI-3-P in σ1B −/− endosomes is indeed due to hyperactive Vps34.

### Rab5/Vps34-pathway is activated by σ1A

**Figure 2:**
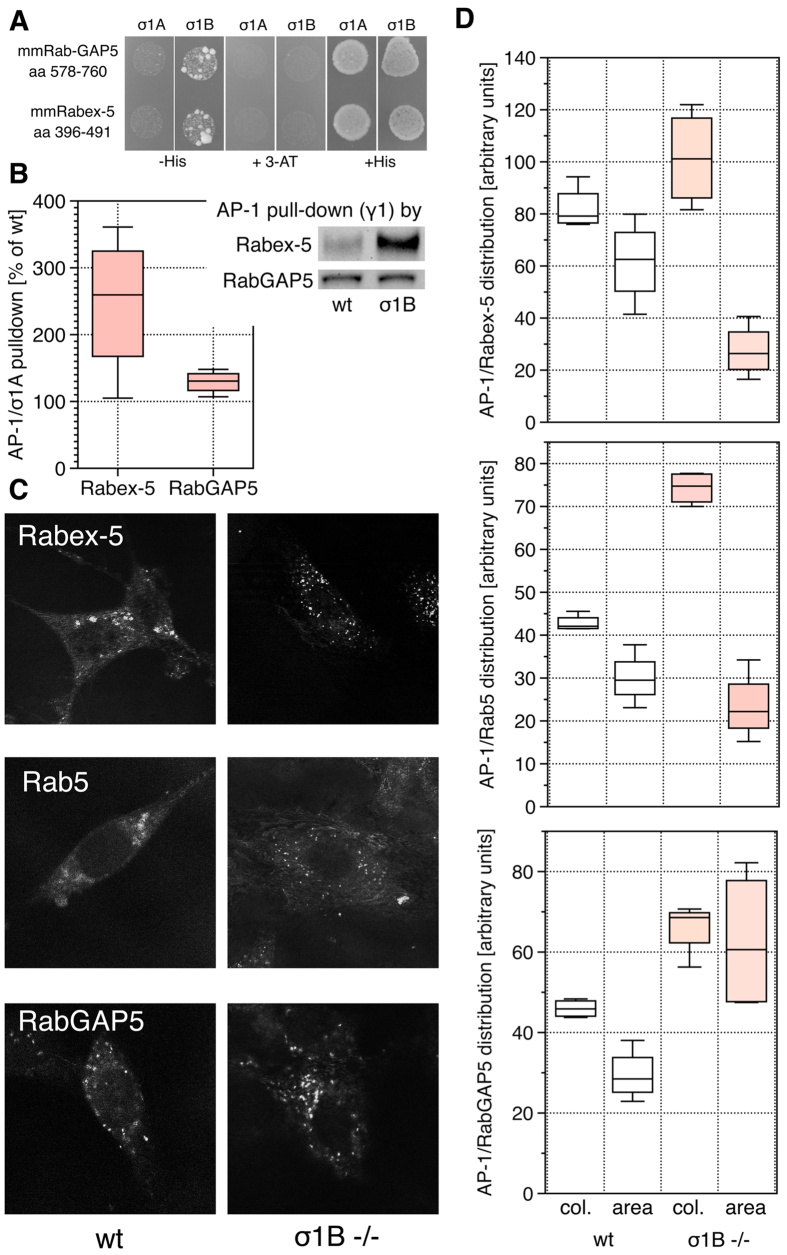
Formation of AP-1 - Rab5 effector-protein complexes. **(A)** σ1 - Rabex-5 and -RabGAP5 binding specificities in the Y3H assay. **(B)** Comparison of AP-1 pulldown by Rabex-5 and RabGAP5 from wt and σ1B −/− brain extracts (n = 4 each). **(C)** PLA assay of AP-1 complex formation with Rabex-5, Rab5 and RabGAP5 as well as their distribution on endosomes (representative IFM) and (**D)** the IFM quantification, col.: amount of AP-1 (γ1-adaptin) colocalisation, area: size of endosomal membrane domain covered by the proteins (cells n = 10 per sample). Comparing the differences between ‘ko’ and wt data sets gave in all χ^2^ tests distributions ≤ 0.004 (>95% probability).

Vps34 PI 3-kinase activity^18^ is stimulated by complex formation with the protein kinase Vps15^25^. The Vps34 C-terminal helical domain blocks the Vps34 active site. Vps15 binding to this helix and the preceding domain stabilises the active-site open conformation of Vps34^20^. Because σ1A and σ1B differ in their C-terminal helical domains, a differential Vps34 binding via these domains could cause the differences in Vps34 activity. σ1A binding could stimulate, or σ1B binding could inhibit Vps34 activity. Neither the Vps34 C-terminal nor the catalytic domain bound σ1-adaptins in γ1/σ1 hemicomplex Y3H assays, suggesting an indirect regulation by σ1 adaptins (Fig. S1). Membrane-cytoplasm recycling of Vps34/Vps15 is a mechanism of Vps34-pathway regulation, but not in neurons, where Vps34/Vps15 remains membrane bound^17^. Rab5^GTP^ binds Vps15, enhancing Vps34 activity by an unknown molecular mechanism^17^, but the amount of Rab5 on σ1B −/− endosomes was not increased^16^. Rab5 cytoplasma-endosome recycling could be reduced in these synaptic endosomes compared to fibroblastoid endosomes, as is the Vps34/Vps15 recycling. Alternatively, other Rab5 functions and the respective GEFs could be reduced, whereas the Vps34-pathway is activated and thus the amount of endosome bound Rab5 would not be altered. Indeed, σ1B −/− early endosomes do contain more of the Rab5-GEF Rabex-5, whereas the neighbouring high density, late endosome fraction contains less^16^. Thus σ1A binding to Rabex-5 could be a mechanism for Rab5-pathway activation. We tested the Rabex-5 C-terminal aa 396–491 for σ1-binding in γ1/σ1 hemicomplex Y3H binding assays, because these mediate Rabex-5 early endosome binding independent of the Rab5/Rabex-5 linker Rabaptin-5α^26^,^27^. Unexpectedly, Rabex-5 binds σ1B, not σ1A (Fig. 2A). In addition, we tested the Rab5-pathway-inactivating RabGAP5 for σ1 isoform binding, because its inhibition could contribute to Rab5-pathway activation. RabGAP5 consists of the catalytic TBC domain, a SH3-domain and a C-terminal RUN-domain, present in proteins linked to GTPase functions^28^. Also the RabGAP5 RUN-domain (aa 578–760) binds σ1B, not σ1A (Fig. 2A). Therefore both proteins might use similar motifs and sequence alignment indicated two related motifs in both: **P_E_**A:E_C:L_**L** and **P_L_**Q:K_P:E_Q:G_**V**. We replaced in Rabex-5 the E and L residues following the P in both putative motifs by A. This abolished σ1B binding in Y3H assays (Fig. S2). Rabex-5 proteins with one of the two motifs mutated gave highly variable results in *in-vitro* AP-1 pulldown experiments, indicating that they are unstable proteins *in-vitro* and that both motifs might act in concert to enable σ1B binding. However, wt Rabex-5 and RabGAP5 domains pulled down AP-1 out of wt and σ1B −/− synaptosome extracts, confirming the Y3H protein interaction data. Rabex-5 isolates 160% more AP-1/σ1A complexes out of σ1B −/− synaptosome extracts and RabGAP5 30% more, which was not expected due to the absence of σ1A-Rabex-5 and RabGAP5 binding in the Y3H experiment (Fig. 2B). Because of these contradictory results from the *in-vitro* experiments, we tested for AP-1 colocalisation with these two proteins and with Rab5 on membranes *in-vivo* with the proximity-ligation-assay (PLA). We used wt, σ1B −/− and AP-1-deficient (μ1A −/−) MEF cell lines. Their endosomes are larger than synaptic endosomes, thus providing a higher spatial resolution. Indeed, in σ1B −/− cells more AP-1/Rabex-5 and also more AP-1/Rab5 colocalised. Moreover, these complexes are confined to smaller areas in σ1B −/− cells compared to wt cells, indicating maturing membrane subdomains (Fig. 2C,D). Also more RabGAP5 colocalises with AP-1/σ1A, but these complexes are more dispersed, which should result in reduced RabGAP5 interaction with Rab5, limiting Rab5-pathway inactivation (Fig. 2C,D). Due to the difference in the AP-1/Rabex-5 and AP-1/RabGAP5 complex distributions, their compositions would be expected to be different. We did not investigate the AP-1/RabGAP5 distribution further at this point, because pulldown experiments and the increased Rabex-5 early endosome binding in σ1B −/− neurons demonstrate that it is the most relevant mechanism. All these data are in line with Rab5^GTP^-mediated activation of Vps34 activity and with our previous biochemical analysis, which showed increased Rabex-5 association with early endosomes^16^. There is, however, a discrepancy with the Y3H data, which show σ1B, but not σ1A binding of Rabex-5 (Fig. 2A).

### ArfGAP1 links σ1A and Rabex-5

**Figure 3:**
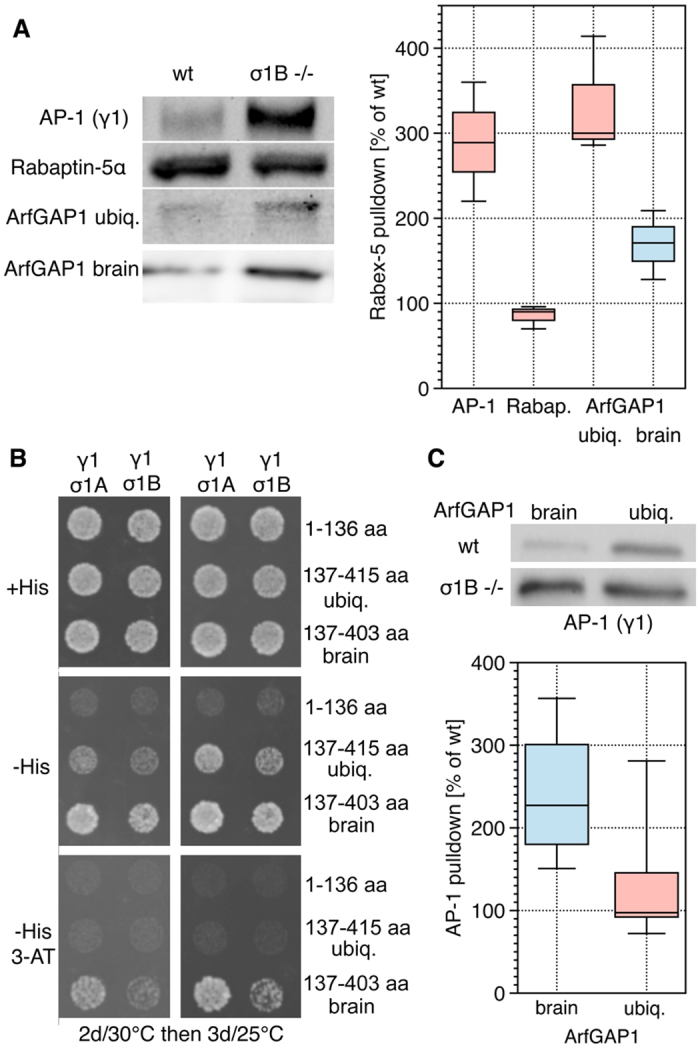
Specificity of AP-1 - ArfGAP1 complex formation. Experiments were performed with extracts from individual animals. (**A)** Isolation of AP-1, Rabaptin-5α and ArfGAP1 isoforms from brain extracts by Rabex-5 pulldowns. Representative Western blots and the quantification (n = 3 each). (**B)** Y3H assay for ArfGAP1-σ1 binding specificities. The GAP-domain (aa 1–136) and the two C-terminal domains of ubiquitous (aa 137–415) and brain-specific ArfGAP1 (137–403) were tested. (**C)** ArfGAP1 isoforms pulldown AP-1 from wt and σ1B −/− brains. Representative Western blots and the quantification are shown (WB, br. n = 4 , ubiq. n = 5). Comparing the differences between ,ko‘ and wt (set to 100%) data sets gave in all χ^2^ test distributions ≤ 1 × 10^−72^ (>99% probability).

The discrepancy between Y3H and pulldown experiments can be explained by the formation of AP-1/σ1A-Rabex-5 complexes by a linker protein, like the Rabex-5/Rab5 linker Rabaptin-5α. Rabaptin-5α is also bound by the C-terminal ‘ear’-domain of AP-1/γ1. Altering the protein ratio of both alters early endosome protein recycling^29^. Thus, we expressed Rabex-5 for pulldown experiments in *E. coli* and incubated the Rabex-5-loaded beads with brain extracts from wt and σ1B −/− mice. Again, more AP-1 complexes were isolated from σ1B −/− brains than from wt brains by Rabex-5 (Fig. 3A). However, Rabex-5 isolated less Rabaptin-5α out of σ1B −/− synaptic extracts than from wt extracts (Fig. 3A), excluding a linker function in this pathway. Thus we tested ArfGAP1 for a σ1A/Rabex-5 linker function for several reasons. Like Rabaptin-5α, it binds the γ1 ‘ear’-domain and it binds to endosomes. Also, it exists as a brain-specific isoform of unkown function^30^,^31^. ArfGAP1 has functions independent of its N-terminal GAP domain activity and the brain-specific isoform has an altered C-terminal domain^32^,^33^,^34^,^35^,^36^. ArfGAP1 stimulates Arf1^GTP^ GTP-hydrolysis by a conformational change induced by binding of its C-terminal lipid-sensor domains, ALPS1 and 2, to highly curved membranes^37^. Arf1^GTP^ recruits AP-1 on membranes and releases it upon GTP-hydrolysis and thus ArfGAP1 regulates AP-1 membrane binding. The brain-specific ArfGAP1 is generated by a deletion/insertion modification altering the ALPS2 motif^31^. Indeed, ubiquitous ArfGAP1^U^ and brain-specific ArfGAP1^B^ were enriched in Rabex-5 pulldowns from σ1B −/− synaptosome extracts compared to wt extracts (Fig. 3A). Therefore we tested for ArfGAP1 binding by σ1 adaptins. It has been shown that the C-terminal ArfGAP1 domain of the ubiquitous isoform binds the γ1 ‘ear’-domain^30^. This interaction did not interfere with our analysis of σ1 isoform binding, because in the Y3H-assays γ1 is expressed without its C-terminal ‘ear’ domains (Fig. 3B). The ArfGAP1^U^ C-terminal domain showed comparably weak, whereas ArfGAP1^B^ showed stronger binding towards σ1A. As expected from the efficient isolation of AP-1/σ1A by Rabex-5 in pulldown experiments (Fig. 3A), both ArfGAP1 C-terminal domains showed significantly lower affinity towards σ1B (Fig. 3B). Thus, we tested, whether C-terminal ArfGAP1 domains, like the Rabex-5 domain, isolate more AP-1/σ1A out of σ1B −/− than from wt synaptosomes. Both isolated AP-1, and specifically ArfGAP1^B^ isolated more AP-1/σ1A out of σ1B −/− synaptosomes (Fig. 3C). This differential isolation would not be possible, if the C-terminal ArfGAP1 domains would bind the γ1 ‘ear’ domain with high affinity, because γ1 is present in both AP-1 complexes. Collectively, these data indicated the formation of AP-1/ArfGAP1/Rabex-5 complexes.

**Figure 4:**
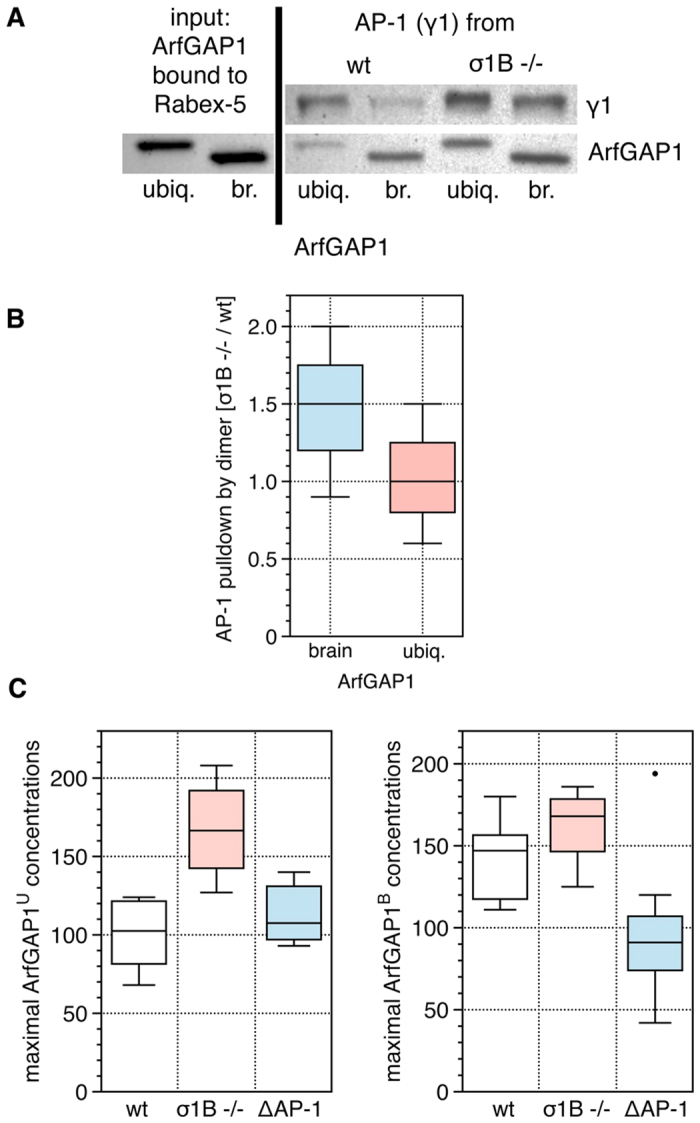
Formation of AP-1/ArfGAP1/Rabex-5 complexes. (**A,B)** *In-vitro* formation of tripartite AP-1/ArfGAP1/Rabex-5 complexes. *E. coli*-expressed Rabex-5 was used to isolate *E. coli* expressed ArfGAP1 brain and ubiquitous isoforms (left panel of representative WB). These Rabex-5/ArfGAP1 complexes are able to isolate AP-1 complexes (anti-γ1 WB) from wt and σ1B −/− synaptosome extracts, which demonstrates an ArfGAP1 linker function. (**B)** Experiments were repeated with extracts from 3 individual animals per genotype and their quantitation is shown (WB, n = 3). The difference between ArfGAP1 isoforms appears to reproduce the differences in σ1 isoform affinities also in the consecutive pull-down experiments, however the χ^2^ test gave p = 0.8 or 55% and more experiments would have to be done. (**C)** AP-1 dependence of the endosomal concentrations of ArfGAP1 isoforms. ArfGAP1^U^ (ubiquitous) and ArfGAP1^B^ (brain) were expressed as GFP-tagged proteins in the MEF cell lines (wt = wild-type; σ1B −/−; ΔAP-1 = μ1A −/− (see TGN concentrations in suppl.) (cells n = 10) Box-plot diagram shows the median and the values outside the 50% percentile as lines; black dot indicates an experiment not included for the statistics. Comparing the significance of the data sets between wt and the mutant cell lines by χ^2^ tests gave distributions ≤ 1 × 10^−16^ (>99%).

To test whether Rabex-5 binds ArfGAP1 proteins and whether such complexes are able to bind AP-1 complexes, *E. coli-*expressed Rabex-5 was first tested for isolating *E. coli*-expressed ArfGAP1 and then those complexes were tested for AP-1 isolation out of brain extracts. Rabex-5 bound both ArfGAP1 proteins (Fig. 4A) and indeed, Rabex-5/ArfGAP1^U^ and Rabex-5/ArfGAP1^B^ complexes isolate more AP-1/σ1A from σ1B −/− synaptosomes (Fig. 4A,B). Even the difference between the ArfGAP1 isoforms appears to be reproducible in these demanding consecutive pull-down experiments, but this is not statistically significant (χ^2^-test distribution 0.8). Thus tripartite AP-1/ArfGAP1/Rabex-5 complexes can form and therefore we tested whether the ArfGAP1 distribution on membranes depends on AP-1 complexes as does the distribution of Rabex-5/Rab5 (Fig. 2C,D). We expressed GFP-tagged ArfGAP1^B^ and ArfGAP1^U^ in wt, σ1B −/− and in MEF cells lacking both AP-1 complexes (μ1A −/−, ΔAP-1)^10^ and determined their distribution on peripheral endosomes and the peri-nuclear trans-Golgi-network (Figs 4C and S3). AP-1 and ArfGAP1 localise to neighbouring domains with only limited colocalisation in wt cells, best visible on the larger trans-Golgi network (TGN), in line with transient interactions (Fig. S3). There was no qualitative difference in the AP-1-dependence of their distributions on both compartments, but the effect was more pronounced on the larger TGN. Over 85% of ArfGAP1 proteins bound endosomes (Fig. S3). Indeed, concentrations of ArfGAP1^U^ and ArfGAP1^B^ increased in the absence of AP-1/σ1B compared to wt cells. They also decreased in ΔAP-1 (μ1A −/−) cells lacking both AP-1 complexes (Fig. 4C). The ArfGAP1^B^ distribution appears to depend more on the presence of AP-1 complexes than ArfGAP1^U^, because its concentration fell below wt levels in ΔAP-1 cells. The ArfGAP1^U^ concentration decreased only to wt levels, when both AP-1 complexes were absent. The second of the two lipid sensor motifs of ArfGAP1^U^, which is modified in ArfGAP1^B^, thus appears to mediate either a lipid-domain dependent distribution^38^ or the association with a yet unknown protein, making its concentration less AP-1-dependent. Nevertheless, ArfGAP1^U^ is also more concentrated, when AP-1/σ1B is absent.

That ArfGAP1 performs this linker function was not expected, because σ1B −/− endosomes contain less ArfGAP1 than wt endosomes^16^. ArfGAP1 has low binding affinity towards low curvature membranes^39^ and thus less ArfGAP1 might bind constitutively to these enlarged endosomes^7^, whereas the remaining ArfGAP1 pool is retained in these complexes with AP-1. Its Arf1^GTP^ GAP-activity is expected to be inhibited in this tripartite endosomal complex^37^, preventing AP-1 membrane dissociation. This explains the increase in AP-1/σ1A complexes bound to σ1B −/− endosomes relative to the reduced amounts in synapses and in the synaptic CCV pool^16^. There is also a marked increase of ArfGAP1 proteins in the endocytic AP-2 CCV pool of σ1B −/− synapses, indicating that their redistribution between these two membrane pools might also be regulated^16^.

### σ1B inhibits Rab5/Vps34-pathway activation by σ1A

**Figure 5:**
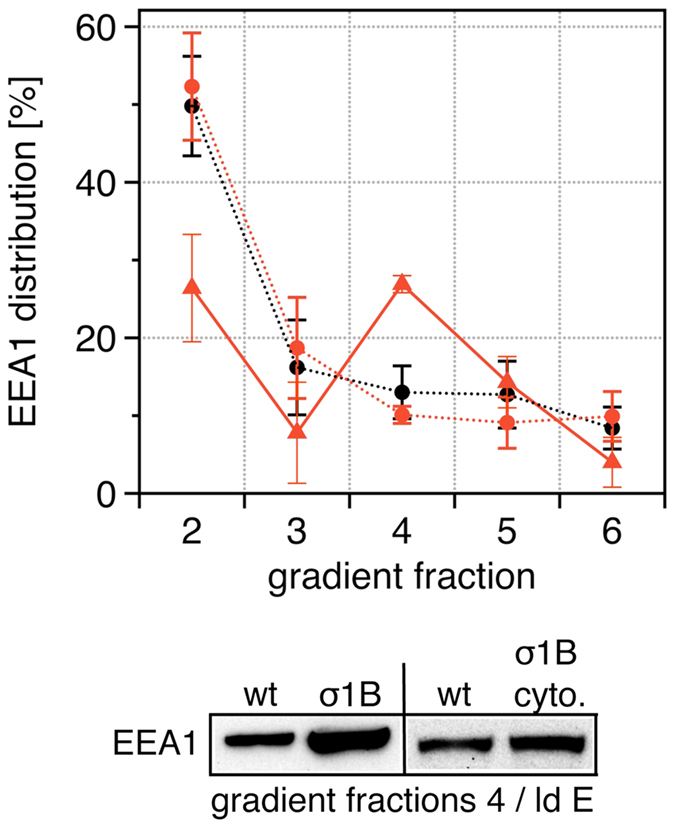
Rescue of the Rab5/Vps34-pathway activation in σ1B −/− brain endosomes. Inhibition of Vps34 activity in σ1B −/− early (low-density; density gradient fraction 4) and late (high-density; density gradient fraction 5) endosomes (as in Fig. 1) by the addition of AP-1/σ1B cytosol to σ1B −/− membranes. PI-3-P was determined by membrane association of the PI-3-P binder EEA1. wt black circles, dotted line (n = 4, s.d.) , σ1B −/− red triangles, solid line (n = 6; s.d.), σ1B −/− plus AP-1/σ1B red circles, dotted line (n = 4, s.d.). Gradient fraction 1 corresponds to the load and it is not included. Representative western-blots of fractions 4, (low-density, early endosomes) are shown. In all experiments wt and σ1B −/− extracts were processed in parallel and compared and thus EEA1 of wt fraction 4 (ld E) is shown twice. χ^2^ test distributions for the data obtained from σ1B −/− extracts for this fraction is 1 × 10^−10^ (>99%).

The formation of AP-1/σ1A/ArfGAP1/Rabex-5 complexes (Figs 2, 3, 4) and the increase in endosomal Rabex-5 in σ1B −/− synapses^16^ indicates that AP-1/σ1B binding to Rabex-5 inhibits formation of the AP-1/σ1A/ArfGAP1/Rabex-5 complexes and thus Rabex-5 recruitment to endosomes. As a consequence the Rab5/Vps34 pathway is less active in wt than in σ1B −/− synapses. To test whether we can rescue the Vps34 hyperactivation on these endosomes by AP-1/σ1B, we added wt cytosols to the density centrifugation. Adding cytosolic proteins to the density gradient solutions enables their interaction with membrane proteins during the entire 5 h long centrifugation, during which synaptic early endosomes are separated from the majority of the endosomes (and normally cytosolic proteins) found in fractions 1 and 2. Brain cytosol was prepared from isogenic wt animals and MEF cytosol was prepared from an isogenic wt cell line. MEF cells contain much less AP-1/σ1B and thus served as negative control^7^. 7 mg protein of σ1B −/− brain extract was loaded on top of the optiprep density gradient (fraction 1). A total of 1.4 mg cytosolic proteins were added to the density gradient solutions before the density gradient was formed. Fraction 4, enriched in synaptic early endosomes, contains 4% of the loaded σ1B −/− brain proteins^16^. The amount of wt cytosolic proteins added also corresponds to 4% (280 μg/gradient fraction) of loaded σ1B −/− proteins per gradient fraction. Vps34 activity and PI-3-P formation was again analysed by EEA1 binding to the endosomal fractions^16^. The increase in PI-3-P seen in σ1B −/− endosomes was inhibited by the addition of wt brain (Fig. 5), but not by wt MEF cytosol (not shown). Collectively, these data strongly suggest that AP-1/σ1B inhibits Vps34 activation by reducing the amount of Rabex-5 bound to these early endosomes.

## Discussion

In synapses of AP-1/σ1B ‘knock-out’ mice SV recycling is slower than in wt synapses, and large early endosomes are formed on which the remaining second AP-1 complex, AP-1/σ1A, accumulates. This indicates that AP-1 complexes take part in SV reformation from these endosomes^7^,^16^. Such a role would be in line with their protein sorting function in CCV formation, but both, the mechanism and its function in SV recycling have yet to be demonstrated. Here we reveal a novel molecular mechanism and a novel AP-1 function in protein sorting and transport, that is not linked to CCV formation. AP-1/σ1A and AP-1/σ1B complexes bound to synaptic early endosomes regulate their maturation into late endosomes, a process required for SV protein degradation via endolysosomes. These late endosomes are MVB late endosomes^7^,^16^ into which membrane and membrane-associated proteins are targeted after they have been modified with ubiquitin^40^.

**Figure 6:**
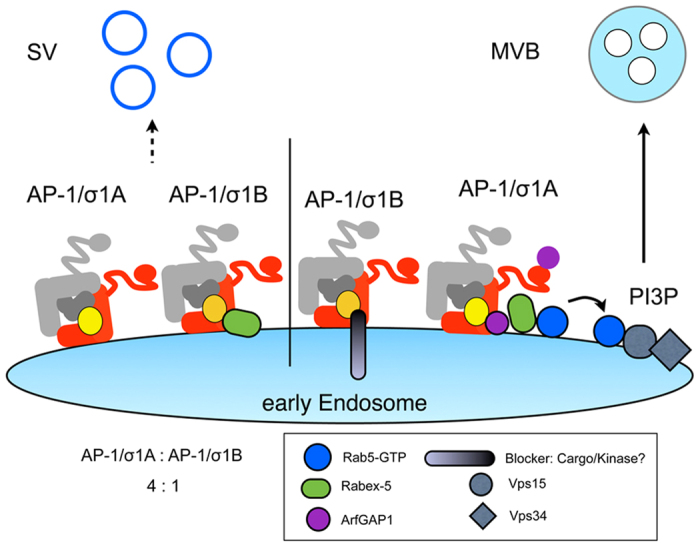
Model for the regulation of SV protein recycling versus SV protein degradation via the multivesicular body late endosome (MVB) pathway by AP-1 complexes AP-1/σ1A and AP-1/σ1B. The Rab5/Vps34-dependent MVB pathway is activated by the ArfGAP1-mediated recruitment of Rabex-5 into a stable AP-1/σ1A - ArfGAP1 complex. Rabex-5 binding to σ1B prevents complex formation and thus stable Rabex-5 recruitment onto endosomes. Post-translational modifications like protein phosphorylation are likely involved in the differential regulation of the pathway by both AP-1 complexes (see also discussion). ArfGAP1 binding to the γ1 (subunit shown in red) ‘ear’-domain may support ArfGAP1 recruitment. σ1A shown in yellow, σ1B in gold, μ1A in dark grey and β1 in light grey.

Endosomal AP-1/σ1A activates the Rab5/Vps34-pathway essential for early endosome maturation into MVB late endosomes via binding of ArfGAP1/Rabex-5 complexes and it does so preferentially with the brain-specific isoform of ArfGAP1. AP-1/σ1A - ArfGAP1 - Rabex-5 complex formation leads to more Rabex-5 bound to these endosomes^16^ and to the concentration of Rabex-5 and Rab5 in AP-1-coated endosomal subdomains. ArfGAP1 stimulates Arf1^GTP^ hydrolysis, leading to membrane dissociation of AP-1. GAP-activity is stimulated by conformational changes in the C-terminal domain^37^. It is thus reasonable to assume that simultaneous binding of σ1A and Rabex-5 to the C-terminal domain prevents activation of the GAP-activity, leading to stable AP-1/σ1A membrane binding. This would explain why endosomes of σ1B −/− synapses contain more AP-1/σ1A complexes than wt endosomes^16^. AP-1/σ1B interferes with formation of this stable complex by binding Rabex-5 directly (Figs 2,3 and 4). Early endosomes from wt cells contain less Rabex-5 and less AP-1/σ1A complexes than σ1B −/− early endosomes^16^ (Fig. 6). Thus, without trapping and inhibiting ArfGAP1, an AP-1/σ1B/Rabex-5 endosomal complex is not stable. In vesicular protein sorting high affinity AP-1 membrane binding requires PI-4-P, a membrane-bound cargo protein and Arf1^GTP^. The instability can be due to low membrane binding affinity of this bipartite complex and to the Arf1^GTP^ inactivation by ArfGAP1. Importantly, σ1B −/− endosomes contain less ArfGAP1 than wt endosomes^16^ and thus ArfGAP1 might be limiting for Arf1^GTP^ activation, but regulatory protein modifications might be involved as well (see also below).

Besides the ArfGAP1-σ1A interaction via the ArfGAP1 C-terminal domain, this ArfGAP1 domain also binds the γ1 ‘ear’-domain^30^. This γ1 globular domain is connected via a long flexible hinge domain to the N-terminal core of γ1, which interacts with σ1 and β1 adaptins. This interaction mode could therefore support ArfGAP1 recruitment to AP-1-coated membranes (Fig. 6). The differential interaction of AP-1/σ1A and AP-1/σ1B complexes with ArfGAP1/Rabex-5 (Figs 2B and 3A) indicates that the ArfGAP1- γ1 ‘ear’ interaction is of comparably lower affinity.

Clathrin is not only involved in CCV formation, but also in the MVB pathway. EM images of σ1B −/− synapses indicated an increase of clathrin on early endosomes, which would be in line with the upregulation of the MVB pathway^7^. However, this early endosomal clathrin pool is not recruited by the early endosomal AP-1 complexes. The ESCRT-0 component Hrs recruits these clathrin molecules and this interaction is essential for the formation of the internal vesicles of MVB endosomes^41^,^42^,^43^.

Besides having GDP-GTP exchange activity, Rabex-5 is also a ubiquitin E3-ligase and thus could also ubiquitinate AP-1/σ1A-bound cargo proteins. This would send those proteins immediately into the Vps34-dependent MVB pathway, which should increase the fidelity and speed of the transport of synaptic proteins into the degradation pathway. Rabex-5 is not the only ubiquitin ligase in synapses and all depend on the activation of the Rab5/Vps34 pathway for delivery of their specific target proteins to MVB endosomes, and not only proteins bound to AP-1/σ1A will be sent into this pathway^44^. By microscopy, Vps34 and its product PI-3-P (FYVE-GFP staining) have been detected in dendrites, but in axons and synapses only trace amounts of Vps34 were detected and PI-3-P was not detected at all^45^,^46^. This indicates that synaptic PI-3-P turnover is especially fast and thus coupled to SV recycling or that the Vps34/MVB-pathway has a highly selective function in synapses, transporting only a limited subset of proteins into MVB endolysosomes for degradation. This is in line with our data demonstrating that not all SV proteins are reduced in σ1B −/− synapses^16^.

We propose a model in which both AP-1 complexes regulate SV reformation as well as SV protein degradation (Fig. 6). The next question we have to answer is the regulation of the balance between SV protein recycling and endosomal transport, and in which context SV protein degradation is induced. We did not succeed in generating σ1-isoform specific antibodies and can only estimate σ1A:σ1B ratios based on the 25% reduction in AP-1 CCV in σ1B −/− synapses^16^. A 4:1 ratio indicates that endosomal AP-1/σ1A regulates also the basal rate of synaptic early endosome maturation, which can be upregulated by the inhibition of σ1B-Rabex5 binding. All proteins identified to be involved are modified by post-translational modifications, and it is likely that complex formation is regulated by modification of one or more of these proteins. Rabex-5 is phosphorylated at its C-terminus on Ser/Thr and Tyr residues (summarised on PhosphoSitePlus), which might prevent binding by ArfGAP1 and thus its stable endosome association. Also ArfGAP1 is modified by phosphorylation. LRRK2 (leucine-rich repeat kinase 2) activity, whose mutations are the most common cause for Parkinson’s Disease, inhibits ArfGAP1 GAP-activity and ArfGAP1 is able to stimulate LRRK2 activity^47^,^48^. LRRK2 has also been shown to be involved in the regulation of SV recycling as well as in late endosome transport^49^,^50^. Therefore LRRK2 is part of the regulatory network, that coordinates protein and membrane traffic in synapses and is thus a candidate kinase for taking part in the regulation of this AP-1 function. We are currently working to identify protein modifications and the respective kinases involved in the regulation of this pathway. In Alzheimer’s disease, like in Parkinson’s disease, disturbed endosomal protein sorting contributes to disease development, and the novel AP-1 function presented here might also play a role in the development of this disease^51^.

In σ1B −/− synapses only a subset of SV proteins is degraded, presumably by their selective modification by E3-ligases, and thus the protein composition of the SV formed should be altered. This should affect properties such as their mobility and their targeting to and anchoring at active zones, as well as their fusion kinetics with the plasma membrane^52^,^53^,^54^. In contrast, the homologous AP-3 complex involved in SV protein sorting during SV biogenesis facilitates direct fusion and degradation of entire SV with Rab7 late endosomes and their degradation^55^,^56^. This pathway bypasses the Rab5 early endosomes and gives a hint why synapses contain more Rab7 than Rab5^57^. The degradation of only a subset of SV proteins in σ1B −/− synapses indicates that the alteration in the composition of the pool of SV proteins are part of an adaptation mechanism. We will test how these changes in σ1B −/− synapses affect SV properties and synapse functions using high resolution microscopy and electrophysiology. The mouse ‘knock-out’ phenotype demonstrates that the AP-1/σ1A - AP-1/σ1B competition in the regulation of early endosome maturation and SV protein degradation is part of a network regulating synaptic plasticity and synaptic silencing essential for the regulation of learning, memory formation and memory recall.

The AP-1/σ1A and AP-1/σ1B complexes regulate the maturation of synaptic early endosomes into late, MVB endosomes. They do so by complex formation with or without ArfGAP1 and Rabex-5. This novel function of the clathrin AP-1 adaptor-protein complexes raises their role to one of the major coordinators of protein export routes out of early endosomes.

## Methods

### Endosome isolation, western blotting, EM images, data analysis

The σ1B mouse ‘knock-out’ model has been described in Glyvuk *et al.* 2010 and has a SV129/BL6 genetic background. Animals used for tissue isolations were −/− animals and isogenic +/+ animals derived from +/− matings. Animals are kept in the central animal facility of the Faculty of Medicine of the Georg-August-University Göttingen in accordance with the appropriate guidelines. Animals were killed with CO_2_ and cervical dislocation in accordance with the appropriate guidelines. Animal housing and the protocol for killing the animals were approved by the ‘Niedersächsisches Landesamt für Verbraucherschutz und Lebensmittelsicherheit’ (LAVES). Brains were immediately isolated and frozen in liquid nitrogen and stored at −80 °C. Differential and OptiPrep^TM^ density centrifugations were done as established and described in detail in Kratzke *et al.*^16^. Membrane bound and soluble proteins were separated by differential centrifugation. Mouse cortex was homogenised with a 1 mL glass homogeniser (10 strokes loose, 10 tight plunger) and centrifuged at 1000 g, 10 min. Supernatant (S1) is named cortex fraction. Centrifugation of S1 at 13000 g for 15 min yielded supernatant S13 and pellet P13. Centrifugation of S13 at 100000 g for 45 min yielded a supernatant S100 and a pellet P100. All steps were performed in 38 mM potassium aspartate, 38 mM potassium glutamate, 38 mM potassium gluconic acid, 20 mM MOPS, 5 mM reduced glutathione, 10 mM potassium carbonate, 0,5 mM magnesium carbonate, 1 mM EDTA, 1 mM EGTA, 1:5000 protease inhibitor cocktail (Sigma, München, Ger), 10 nM Calyculin A, 1 mM Na_3_VO_4_, pH 7,1) at 4 °C. Continuous OptiPrep™ (Axis-Shield, Heidelberg, Ger) gradients were prepared using a Gradient Station ip (BioComp Instruments, Fredericton, CA). Cortex fractions were prepared as described above. Synaptosomes were lysed by 20 passages through a ball homogeniser (Isobiotec, Heidelberg, Ger) with a clearance of 12 μm. This extract was layered over 0–30% OptiPrep™ gradients, centrifuged at 65000 g for 5 h and six fractions were collected. Protein concentration was determined according to the Bradford assay (BioRad, Munich, Ger). Fractions of the velocity centrifugation were mixed with 60% OptiPrep™ to a final concentration of 32.5% or greater, laid under a continuous 0–30% OptiPrep™ gradient and centrifuged at 100000 g for 18 h. For the complementation experiment 7 mg of σ1B −/− brain lysate was loaded on top of the preformed gradient (fraction 1). 1.4 mg of wt cytosolic proteins (100.000 × g supernatant) was mixed in the gradient density buffers before the gradient was formed (fractions 2–6). The EEA1 antibody was sc-6414 (Santa Cruz, USA) and the Vps34 antibody was #3811 (Cell Signaling, USA). Secondary, HRP-conjugated antibodies were from Dianova (Hamburg, Germany). Western blots were developed using luminescence reagents (Millipore) and images were recorded using the Fuji LAS 1000 (Fujifilm Corp., Düsseldorf, Germany). The protein content in each fraction is expressed in % of the total. Diagrams were generated using DataGraph (Visual Data Tools, USA). Box-plot diagrams show the distribution of all the data and their median and the values outside the 50% percentile as lines. Data not included for the calculation of the median are indicated by a black dot. Each data set is based on a minimum of 3 independently performed experiments. The significance of the differences detected in experiments comparing data from wt and σ1B −/− mice as well as data obtained with different protein isoforms were verified by the χ^2^ (chi square, Excel) test, because it is most appropriate for smaller sample numbers. In addition, it allows to compare ‘knock-out’ with wt data sets, which were defined as 100% signal intensity in each experiment. χ^2^ distributions ≤ 0.004 indicate a ≥ 95% probability (m = 1) that two data sets are different. EM images of the gradient fraction enriched in synaptic endosomes were prepared after concentration of the gradient fractions from 400 μl to 50 μl and glutaraldehyde fixation and uranyl-actetate staining following standard EM procedures.

### Yeast-3-hybrid assays

γ1 N-terminal core domain (1–550 aa) encoding murine cDNA was cloned into pGADT7 (GAL4 DNA-AD-fusion). Murine σ1-adaptin ORFs were cloned in the MSC-II of pBridge and the indicated proteins and protein domains to be tested for σ1-binding were cloned into the MSC-I site (GAL4 DNA BD-fusion) of pBridge (InVitrogen, Karlsruhe, Germany). Plasmids were transformed for growth and interaction assays into the yeast strain AH109.

### Rabex-5 and ArfGAP1 pulldown

The Rabex-5 C-terminal aa396–491 domain and RabGAP5 C-terminal aa578–760 encoding cDNAs were cloned into pGEX4T1 and expressed with an N-terminal GST-tag. ArfGAP1 protein domains were expressed as N-terminal 6His-tagged fusions from pKM260 in *E. coli* BL21D3 strain (0.5 mM IPTG 4 h, 37 °C). Cells were lysed in PBS buffer (140 mM NaCl, 2.5 mM KCl, 6.5 mM NaHPO_4_, 1.5 mM KH_2_PO_4_, 1 mM PMSF, pH 7.4; 30 min, Lysozyme 0.1%, Proteinase Inhibitors; sonicated 3 min at 4 °C). After centrifugation at 4,000 rpm, 25 min, 4 °C, pellet was resuspended in PBS with 7 M Urea, sonicated 3 min, 4 °C to dissolve inclusion bodies. Bacterial lysates were incubated at 4 °C overnight with Gluthatione Sepharose Beads (Amersham Bioscience) or with Ni-NTA agarose Beads (Qiagen). Resins were harvested and washed with 30 volumes PBS buffer 5 times. Brains were sliced in 1.5 mL PBS pH 7.4, proteinase inhibitors (Roche), and homogenised (glass potter: 30 strokes loose and then tight piston). Homogenate was centrifuged, 1000 g, 10 min, and supernatant (S1) was decanted in a tube, the pellet (P1) was washed and centrifuged, 1000 g, 10 min; the supernatant (S2) was added to S1 and centrifuged at 9200 g, 15 min. The supernatant (S3) was discarded and the pellet (P3) resuspended in 1,5 mL and centrifuged: 10200 g, 15 min. Pellet (P10) was resuspended in 500–600 μL of buffer and homogenised with a ball homogeniser: clearance 12 μm (Isobiotec, Heidelberg, Germany), 40 passages. Homogenised synaptosomes were centrifuged, 25000 g, 20 min. Supernatants were incubated with Glutathione- or nickel-beads respectively for 4 hours at RT. Beads were harvested and washed with PBS 5 times and loaded on SDS-PAGE.

### *In-vivo* distribution of proteins

MEF cell lines used have been established in the literature cited in the results. Cells were fixed with 4% PFA, AP-1 was labelled with anti-γ1 antibody (BD 610386) and Rab5 (Abcam ab18211) or Rabex-5 or RabGAP5 (Proteintech 12735-1-AP, 20825-1-AP) antibodies. Subsequent steps of the ‘proximity-ligation-assay’ were done according to the manufacturer instructions (Sigma). pEGFP-C2 ArfGAP1^U^ and ArfGAP1^B^ plasmids were transiently transfected into MEF cell lines. Cells were fixed in 4% PFA, AP-1 was labelled with anti-γ1 (BD biosciences) and secondary Alexa 633 antibodies (life technologies). Confocal images were recorded with a Leica SP2 microscope (Leitz, Germany) and quantified using the microscopes software package and ImageJ software (NIH, USA).

## Additional Information

**How to cite this article**: Candiello, E. *et al.* AP-1/σ1A and AP-1/σ1B adaptor-proteins differentially regulate neuronal early endosome maturation via the Rab5/Vps34-pathway. *Sci. Rep.* **6**, 29950; doi: 10.1038/srep29950 (2016).

## Supplementary Material

Supplementary Information
