# Supplementary material for: AP-1/σ1A and AP-1/σ1B adaptor-proteins differentially regulate neuronal early endosome maturation via the Rab5/Vps34-pathway
Source: Sci Rep. 2016 Jul 14;6:29950. doi: 10.1038/srep29950 (PMC4944158; doi:10.1038/srep29950)
Supplement: Supplementary Information [file srep29950-s1.pdf]

AP-1/σ1A and AP-1/σ1B adaptor-proteins differentially regulate neuronal early endosome maturation via the Rab5/Vps34-pathway

running title: AP-1 regulates endosome maturation

keywords: AP-1, early endosomes, neurotransmission, Rab5, Vps34

Ermes Candiello, Manuel Kratzke, Dirk Wenzel[1], Dan Cassel[2] and Peter Schu*

Georg-August University Göttingen, Department for Cellular Biochemistry, Humboldtallee 23, D-37073 Göttingen, Germany. [1]Electron microscopy, Max-Planck-Institut for Biophysical Chemistry, Am Fassberg 11, D-37077 Göttingen. [2]Israel Institut of Technology, Department Biology, Haifa 32000, Israel. *corresponding author, pschu@gwdg.de.

Supplementary Information

Supplemental Information

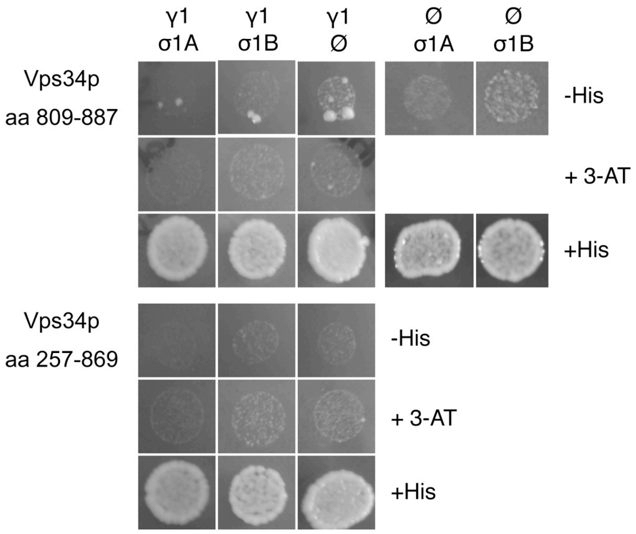

Figure S1: AP-1/Vps34p direct interactions: Determination of σ1-adaptin binding of Vps34p domains by the yeast-3-hybrid assay. The C-terminal, regulatory Vps34p domain (aa 809-887) showed weak, unspecific binding to the N-terminal core γ1 adaptin domain, but no binding to either σ1A or σ1B. This weak interaction is not due to an autoactivation, because without γ1, the weak binding activity was lost. We did not analyze this weak binding in more detail, because it can not explain σ1 isoform specific functions. This binding could also be mediated by a γ1 domain occupied or sterically blocked by β1 and μ1 adaptins. The core domain of Vps34p (aa 257-869), which contains the catalytic center, did not bind to any of the adaptins. In the presence of the auxotrophic marker histidine (+His) all yeast clones grow, excluding a toxic effect of the non-yeast proteins and protein fragments. Addition of the histidine-synthesis blocker 3-AT to 2.5 mM inhibits growth of the yeast clones, confirming that they are indeed auxotrophic for histidine.

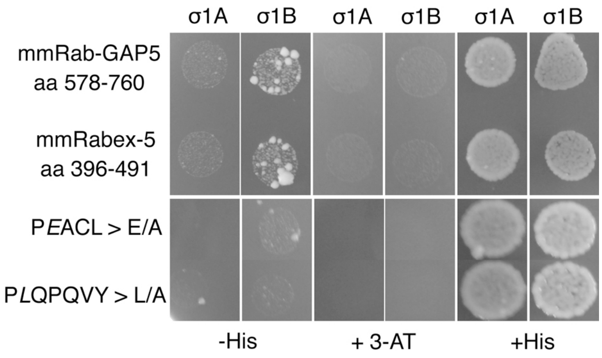


```
 Rabex-5 390  PRKQESESWP PEACLGVKQM YKNLDLLSQL NERQERIMNE AKKLEKDLID WTDGIAKEVQ
Rab-GAP5 618  RLDEDGKVLT PEELLYRAVQ SVNVTHDAAH AQMDVKLRSL ICVGLNEQVL HLWLEVLCSS

 Rabex-5 -491 DIVEKYPLEI KPPNQPLAAI DSENVENDKL PPPLQPQVYA G
Rab-GAP5 -740 LPTVEKWYQP WSFLRSPGWV QIKCELRVLC CFAFSLSQDW ELPARREEEK QPLKEGVQDM
```

Figure S2: Rabex-5 sequence motif for σ1B binding: Sequence alignment of Rabex-5 and RabGAP5 and the effects of amino acid exchanges on the σ1B binding. Rabex-5 and RabGAP5 bound σ1B, but not σ1A adaptin, indicating that both proteins might use the same sequence motif for σ1B binding. Aligning there sequences revealed two homologous motifs in both proteins: **P_E_**A:E_C:L_**L** and **P_L_**Q:K_P:E_Q:G_**V**. Homologous sequence motifs are boxed. Exchange of the E and L residues following the P's in both sequences by A in Rabex-5, indeed abolished σ1B binding in yeast-3-hybrid assays.

A

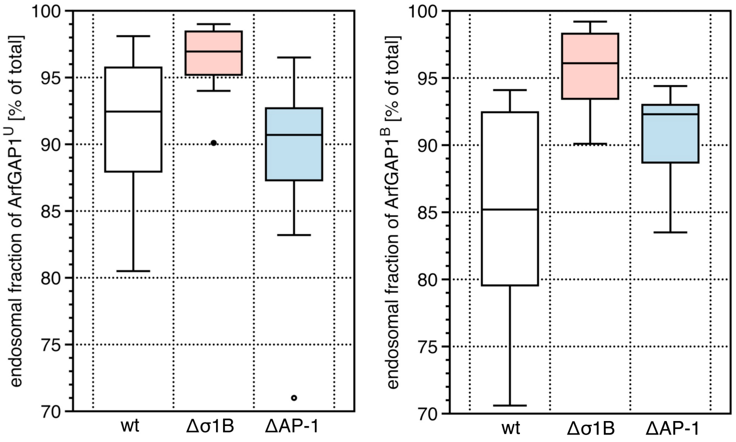
endosomal fraction of ArfGAP1U [% of total]
endosomal fraction of ArfGAP1B [% of total]
100
95
90
85
80
75
70
wt
Δσ1B
ΔAP-1

B

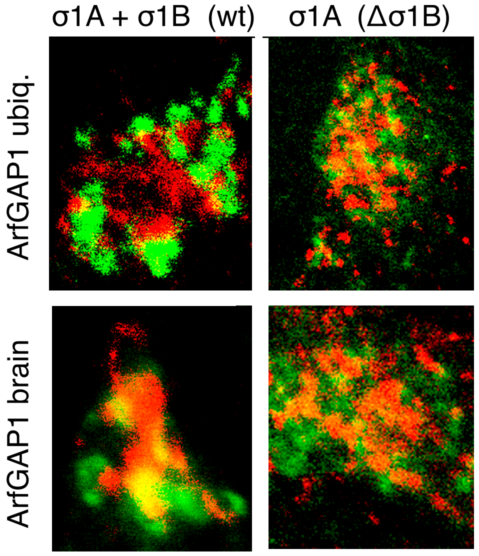
σ1A + σ1B (wt)
σ1A (Δσ1B)
ArfGAP1 ubiq.
ArfGAP1 brain

C

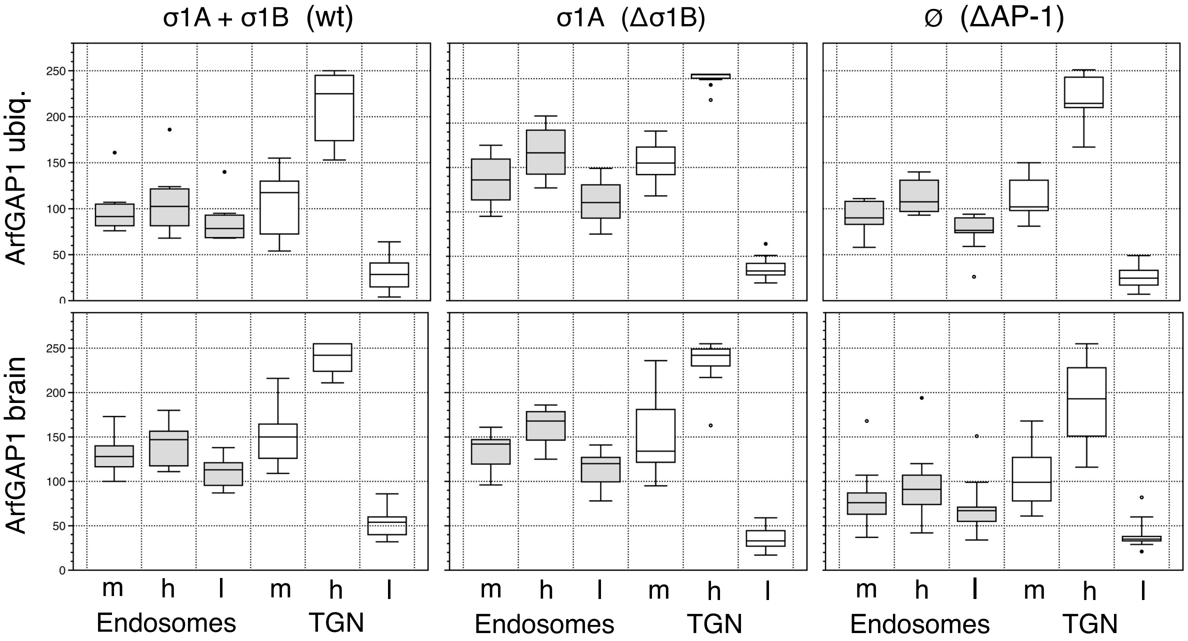
σ1A + σ1B (wt)
σ1A (Δσ1B)
Ø (ΔAP-1)
ArfGAP1 ubiq.
ArfGAP1 brain
250
200
150
100
50
0
m h l m h l
Endosomes
TGN

Fig. S3: AP-1/σ1A and AP-1/σ1B dependency of ArfGAP1 distribution on membranes: **A** We expressed GFP-tagged versions of the ubiquitously expressed ArfGAP1 and of the brain specific ArfGAP1 isoform in mouse embryonic fibroblast cell lines from wt, σ1B -/- and mice deficient in any functional AP-1 complex (μ1A -/-, ΔAP-1) and determined their distribution on peripheral endosomes as well as on the peri-nuclear trans-Golgi network. Over 85% of the ArfGAP1 proteins bound to endosomes and this fraction increased in σ1B -/- cells. Fraction sizes of endosomal GFP-ArfGAP1 proteins in the mouse embryonic fibroblast (MEF) cell lines. **B** Confocal microscopy images showing the localization of AP-1 complexes, labelled by anti-γ1 antibodies and Alexa-633 secondary antibodies (red), and of GFP-ArfGAP1 proteins at the peri-nuclear trans-Golgi network. AP-1 and ArfGAP1 proteins formed neighboring domains with limited colocalization, best visible on the larger trans-Golgi network, in line with transient interactions. **C** Distribution of both ArfGAP1 proteins on endosomes and the trans-Golgi network (TGN) in the various MEF cell lines. Numbers are the signal intensities, expressed as mean (m), highest (h) and lowest (l) intensity ranges of the respective organelles within a cell. There was no qualitative difference in their distribution of both proteins on endosomes and on the trans-Golgi network in the various cell lines. The range of concentrations was much higher in the trans-Golgi network than on endosomes. The trans-Golgi network membrane is larger than the area of the labelled endosomes allowing for a wider distribution of the proteins and indicating the concentration of the proteins in smaller subdomains.

The quantifications shown in A and C were determined in $n \geq 10$ cells and the box-plot diagrams depict the statistics of the cohort.
